# Growth patterns by sex and age among under-5 children from 87 low-income and middle-income countries

**DOI:** 10.1136/bmjgh-2021-007152

**Published:** 2021-11-30

**Authors:** Janaína Calu Costa, Cauane Blumenberg, Cesar Victora

**Affiliations:** 1 International Center for Equity in Health, Federal University of Pelotas, Pelotas, RS, Brazil; 2 Postraduate Program in Epidemiology, Federal University of Pelotas, Pelotas, RS, Brazil

**Keywords:** nutritional status, sex differences, health surveys, socioeconomic factors, age groups

## Abstract

**Introduction:**

Although boys tend to be more affected by linear growth faltering than girls, little is known about sex differences across distinct age groups. We aimed to compare sex differences in linear growth throughout the first 5 years of life among children from low-income and middle-income countries.

**Methods:**

We analysed 87 cross-sectional Demographic and Health Surveys and Multiple Cluster Indicator Surveys (2010–2019). Growth was expressed as height-for-age z-scores (HAZ) based on the 2006 WHO Growth Standards. Sex-specific means were estimated for each country and results were pooled through random-effects meta-analysis for all children and by 12-month age groups. Using linear regression, we assessed the association between sex differences in HAZ and gross domestic product as a proxy for national economic development.

**Results:**

Boys presented lower mean HAZ than girls in the first 30 months. Sex differences were mostly absent between 30 and 45 months, and in several countries, girls had lower HAZ at ages over 45 months. The pooled sex difference (boys minus girls) for the whole sample was −0.10 (95% CI −0.12 to −0.08). The difference was −0.17 (95% CI −0.20 to −0.14) at 0–11 months and −0.22 (95% CI −0.25 to −0.19) at 12–23 months. This was followed by a narrowing of the sex gap to −0.10 (95% CI −0.13 to −0.07) and −0.04 (95% CI −0.07 to −0.01) among children aged 24–35 and 36–47 months, respectively. At 48–59 months, there was evidence of female disadvantage; the mean height-for-age of boys was 0.02 (95% CI 0.00 to 0.05) SDs higher than for girls. Ecological analyses showed that in all age groups, male disadvantage decreased with increasing national income, and this was no longer present for the 4-year-old children, particularly in wealthier countries.

**Conclusion:**

Male disadvantage in linear growth is most evident in the first years, but by the age of 4 years, the sex gap has mostly disappeared, and in some countries, the gap has been reversed.

**Key questions**

**What is already known?**

- Linear growth faltering in early childhood has long-term negative consequences.
- When compared with children growing under optimal circumstances, boys are more likely to present growth faltering than girls.
- Most studies failed to present the results by narrower age groups.

**What are the new findings?**

- Up to about 30 months of age, boys were more likely than girls to present growth faltering in comparison to international standards, but male disadvantage tended to disappear in the fourth and fifth years of life.
- The gap reduced at ages 30–45 months in most countries, and in some, there was evidence of female disadvantage at later ages, however, the magnitude of the differences varied by world’s region.
- The early male disadvantage pattern is most pronounced in the poorest countries.

**What do the new findings imply?**

- Sex differences in child growth vary substantially with the age of children and from one country to another, highlighting the importance of sex-specific and age-specific analyses.
- In early life, male disadvantage reflects the biological frailty of boys, and economic and cultural factors at the country level influence the sex gap in linear growth. Particularly in poor countries, where health and nutrition interventions are insufficient, the gap tends to be wide.
- Our results may support policies and programmes targeting child nutrition and health equity, taking into account the differences between boys and girls over childhood as well as considering geographical, socioeconomic and cultural determinants of care and nutrition.

## Introduction

Globally, undernutrition affects more than 144 million under-5 children, representing a major public health problem.^1 2^ In addition to increasing the risk of short-term morbidity and mortality,^3 4^ undernourished children suffer long-term consequences, including short adult height, reduced intellectual development and economic productivity, and low offspring birth weight.^5 6^

Nutritional deficiency in early life is reflected by poor linear growth, which is usually identified by calculating a standardised difference between children’s heights (or lengths) and age-specific and sex-specific standard values and expressed as z-scores.^7^ The global standard is assumed to reflect the optimal growth of young children, being derived from a study in six countries of children whose families live under best-possible socioeconomic, nutritional, environmental and healthcare conditions. In addition to individual-level growth monitoring, the standard allows the anthropometric assessment of groups of children with different sex and age compositions, being widely used for cross-country comparisons.^8^

Growth faltering, usually identified by prevalence of stunting (height-for-age below −2 z-scores relative to the international growth standard) presents remarkable inequalities over the world. In 2020, more than half of all under-5 children affected by stunting lived in Asia and about 40% lived in Africa.^2^ Socioeconomic factors also account for a large proportion of between-country variability in undernutrition, with stunting prevalence of 34.6% in low-income, 29.1% in lower-middle and 10.8% in upper-middle-income countries.^1 2^

Sex differences in linear growth are well known, with mean height (or length) values for boys being higher than for girls.^7^ Nevertheless, when compared with the international sex-specific standards, boys from several low-income and middle-income countries (LMICs) tend to present a higher prevalence of stunting—or low height (or length) for age—than girls from the same populations.^9^ In other words, when compared with children growing under optimal circumstances, boys are more likely to present growth faltering than girls. This difference has been summarised in a meta-analysis including 38 studies conducted up to 2020 that found higher odds of stunting for boys than for girls with a pooled OR of 1.29 (95% CI 1.22 to 1.37).^9^ Such findings are apparently in contrast with reports that girls face gender discrimination in healthcare, which may result in poor health and possibly in a higher prevalence of undernutrition compared with boys.^10 11^

Although sex differences in the first 5 years of age have been described, most studies failed to break down results by narrower age groups,^9^ with a few exceptions.^12 13^ We compared sex differences in linear growth throughout the first 5 years of life using samples of children from a large number of LMICs, using 1-year age groups. We also assessed whether the sex and age patterns varied according to the region and country income levels.

## Methods

### Data sources

The analyses were based on cross-sectional data from the most recent nationally representative surveys conducted in LMICs between 2010 and 2019 and for which anthropometric measures were available. Demographic and Health Surveys (DHS) and Multiple Indicator Cluster Surveys (MICS) were used, which are designed to provide information on maternal and child health and nutrition using multistage probabilistic sampling processes. Primary sampling units and households were drawn from geographical-based sampling frames that cover the full territory of each country. The study population is made up of samples of children aged 0–59 months born to mothers aged 15–49 years and alive at the time of the interview, including singleton and multiple births.

### Measures

Child growth was measured as sex-specific length or height-for-age z-scores (henceforth referred to as HAZ) according to the 2006 WHO Child Growth Standards. The z-scores represent the difference between an individual child’s value, in SDs, from the median of the reference population (which is standardised and set to 0 with an SD of 1.0). The measures were taken as supine length for children aged up to 23 months and standing height for children aged 24–59 months by trained personnel using infantometers and stadiometers, respectively. The measuring boards used to collect anthropometric measures included ShorrBoards, Seca 217, or locally manufactured boards.

Child age was calculated by subtracting the date of interview from the date of birth reported in the questionnaire and treated as a continuous measure ranging from 0 to 59 complete months. For some analyses, age was treated as a categorical variable, grouped into five 12‐month categories (0–11; 12–23; 24–35; 36–47; 48–59 complete months). Sex was a binary variable (boys/girls).

The WHO Anthro software used for z-score calculations is available at http://www.who.int/childgrowth/software/en/ (WHO Anthro for personal computers, V.3.2.2, 2011: Software for assessing growth and development of the world’s children. Geneva: WHO, 2010). Following the WHO recommendations, standardised scores were considered valid when they were between −6 and 6 SD; values outside this range were discarded.

### Statistical analyses

Based on individual anthropometric measures, from each country database, we derived 120 mean HAZ values for each age in complete months (from 0 to 59), for boys and girls separately. These estimates were calculated accounting for the sample weights and multistage cluster sampling design of the surveys. Next, we regressed HAZ on age by sex using a local polynomial approach weighted for the population of under-5 children from each country, fitting the predicted curve of the association between HAZ and age.

Next, we estimated sex-specific country-level mean HAZ for the following six age groups: all children under 5 years, and the five 12 month age groups described above. Then, the pooled mean HAZ by sex for each age group was estimated across all countries using random-effects meta-analysis, to account for the large heterogeneity between countries’ HAZ estimates, with the restricted maximum-likelihood method.^14^ Based on the pooled means, the difference between boys and girls, expressed in SD, was also calculated in each age group using the same meta-analytical approach.

The meta-analyses were repeated for the seven Unicef regions: East Asia and Pacific (9 countries), Eastern Europe and Central Asia (14), Latin America and Caribbean (12), Middle East and North Africa (7), South Asia (6), Eastern and Southern Africa (17), and West and Central Africa (22).

We described the total number of countries in which the mean HAZ was lower among either boys or girls, for each of the age groups, along with the number of countries in which the sex difference was statistically significant at the 5% level. We fitted linear regression models, which are equivalent to t-tests allowing for weighting and clustering, to estimate p values for the differences.

Lastly, we carried out country-level ecological analyses to assess the association between sex differences in mean HAZ (estimate for boys minus estimate for girls) and per capita gross domestic product (GDP, in current international dollars converted by purchasing power parity factor, natural log) using linear regression. National GDP estimates were obtained from the World Bank data repository,^15^ for the same year as the national surveys. The analyses were performed for all under-5 children as well as for each of the five 12-month age groups.

All analyses were performed using Stata software, V.16.1 (Stata Statistical Software: Release 16, StataCorp).

### Patient and public involvement

No patients or members of the public were involved in the design, analysis or reporting of this study.^16 17^

## Results

A total of 87 LMICs (41 DHS and 46 MICS) had data available on child anthropometric measures with sample sizes that allowed stratification by age in months and sex. The median survey year was 2014 (ranging from 2010 to 2019) and the total number of under-5 children ranged from 786 in Montenegro to 240 098 in India, resulting in a pooled unweighted sample of 824 643 children. A total of 35 710 children were excluded due to missing information. Data sources, survey years, and sample sizes are presented in online supplemental etable 1.

10.1136/bmjgh-2021-007152.supp1 Supplementary data

**Figure 1:**
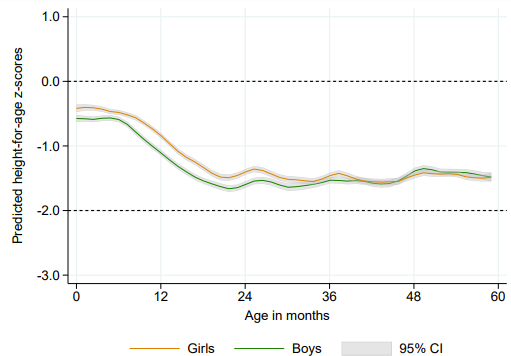
Local polynomial predicted curves for mean height-for-age z-scores according to age in months, by sex. Pooled results for under-5 children from 87 low-income and middle-income countries.

As shown in figure 1, based on data from all countries, the mean HAZ for both girls and boys was below the median reference value for all age groups. The curves are close to the reference during the first months of life, but a steep decline is observed between six and 20 months for both sexes. From 2 to 5 years, mean values in both sexes remained between 1 and 2 SD below the reference. ‘Bumps’ in the growth curves of boys and girls are observed around multiples of 12 months, most likely due to age heaping.

Girls presented a significant advantage over boys up to the age of 30 months, but the male disadvantage was gradually reduced so that from 30 to 45 months there was an overlap of the CIs for both sexes (figure 1). At ages 46–59 months, there was an inversion in the pattern when mean HAZ among boys tended to be higher than for girls, indicating a female disadvantage at older ages. Predicted values by age and the corresponding 95% CIs are presented in online supplemental etable 2.

Table 1 presents sex-specific mean HAZ and sex differences (boys minus girls) for each age group, based on random-effects meta-analyses from 87 LMICs. The pooled mean HAZ for the whole sample of under-5 children was −0.99 for boys and −0.90 for girls, with a significant difference of −0.10 SDs (95% CI −0.12 to −0.08). The age-disaggregated estimates confirm the results shown in figure 1, illustrating the male disadvantage during the first 2 years of life. For children aged 0–11 months, the difference was −0.17 (95% CI −0.20 to −0.14), increasing to −0.22 (95% CI −0.25 to −0.19) for the 12–23 months group. This is followed by a reduction to −0.10 (95% CI −0.13 to −0.07) and −0.04 (95% CI −0.07 to −0.01) among children aged 24–35 and 36–47 months, respectively. The mean difference shows a shift from a male disadvantage to a female disadvantage pattern for children in the oldest group (48–59 months), when the difference among boys and girls was positive (0.02, 95% CI 0.00 to 0.05).

**Table 1:** Mean height-for-age z-scores according to sex and mean sex difference (boys minus girls) by age groups. Pooled results from 87 low-income and middle-income countries.

Age group	Boys		Girls		Sex difference	
	Mean	95% CI	Mean	95% CI	Mean	95% CI
Total (0–59 months)	−0.99	−1.12 to −0.86	−0.90	−1.01 to −0.78	−0.10	−0.12 to −0.08
0–11 months	−0.45	−0.57 to −0.34	−0.28	−0.39 to −0.17	−0.17	−0.20 to −0.14
12–23 months	−1.11	−1.25 to −0.98	−0.90	−1.02 to −0.77	−0.22	−0.25 to −0.19
24–35 months	−1.23	−1.37 to −1.08	−1.13	−1.27 to −1.01	−0.10	−0.13 to −0.07
36–47 months	−1.16	−1.30 to −1.02	−1.12	−1.25 to −0.98	−0.04	−0.07 to −0.01
48–59 months	−1.02	−1.15 to −0.88	−1.05	−1.17 to −0.92	0.02	0.00 to 0.05

In table 2, we present the results according to UNICEF regions. Sex differences were consistent in all regions over the two first years of age (0–11 and 12–23 months), with lower mean HAZ for boys than for girls. For the 24–35 months group, the differences between boys and girls were smaller than for younger children, reaching statistical significance only in Eastern and Southern Africa and in West and Central Africa. No other region showed significant differences, and in Eastern Europe and Central Asia, there was some evidence of female disadvantage. At 36–47 months, the significant female disadvantage was present in Eastern Europe and Central Asia and South Asia. For older children (48–59 months), the female disadvantage was observed in all regions except for the two sub-Saharan African regions.

**Table 2:** Mean height-for-age z-scores according to sex and mean sex difference (boys minus girls) by age groups and UNICEF region

Age group	Region	Boys		Girls		Sex difference	
		Mean	95% CI	Mean	95% CI	Mean	95% CI
0–59 months	East Asia and the Pacific	−1.01	−1.45 to −0.56	−0.92	−1.34 to −0.49	−0.09	−0.13 to −0.05
	Eastern and Southern Africa	−1.47	−1.62 to −1.32	−1.29	−1.44 to −1.15	−0.18	−0.21 to −0.15
	Eastern Europe and Central Asia	−0.15	−0.31 to 0.02	−0.17	−0.33 to −0.01	0.03	−0.02 to 0.08
	Latin America and Caribbean	−0.69	−1.02 to −0.37	−0.62	−0.94 to −0.30	−0.06	−0.1 to −0.02
	Middle East and North Africa	−0.79	−1.31 to −0.27	−0.74	−1.21 to −0.28	−0.04	−0.11 to 0.03
	South Asia	−1.35	−1.56 to −1.14	−1.32	−1.52 to −1.13	−0.03	−0.06 to 0.01
	West and Central Africa	−1.28	−1.41 to −1.15	−1.12	−1.24 to −1.00	−0.16	−0.18 to −0.13
0–11 months	East Asia and the Pacific	−0.34	−0.77 to 0.10	−0.18	−0.57 to 0.22	−0.19	−0.29 to −0.09
	Eastern and Southern Africa	−0.82	−1.02 to −0.63	−0.60	−0.79 to −0.42	−0.22	−0.27 to −0.16
	Eastern Europe and Central Asia	0.18	0.04 to 0.32	0.33	0.23 to 0.42	−0.15	−0.25 to −0.06
	Latin America and Caribbean	−0.31	−0.77 to 0.14	−0.18	−0.60 to 0.23	−0.12	−0.21 to −0.02
	Middle East and North Africa	−0.27	−0.55 to 0.01	−0.14	−0.41 to 0.13	−0.13	−0.21 to −0.04
	South Asia	−0.77	−0.92 to −0.61	−0.64	−0.83 to −0.45	−0.14	−0.20 to −0.08
	West and Central Africa	−0.65	−0.77 to −0.53	−0.45	−0.55 to −0.35	−0.19	−0.24 to −0.15
12–23 months	East Asia and the Pacific	−1.12	−1.57 to −0.67	−0.87	−1.29 to −0.46	−0.23	−0.33 to −0.13
	Eastern and Southern Africa	−1.67	−1.84 to −1.5	−1.36	−1.51 to −1.20	−0.32	−0.37 to −0.27
	Eastern Europe and Central Asia	−0.21	−0.41 to 0.00	−0.14	−0.31 to 0.03	−0.08	−0.15 to −0.01
	Latin America and Caribbean	−0.86	−1.18 to −0.54	−0.61	−0.95 to −0.27	−0.23	−0.32 to −0.15
	Middle East and North Africa	−0.83	−1.36 to −0.30	−0.63	−1.11 to −0.16	−0.20	−0.28 to −0.11
	South Asia	−1.45	−1.63 to −1.27	−1.36	−1.47 to −1.24	−0.16	−0.19 to −0.13
	West and Central Africa	−1.38	−1.49 to −1.27	−1.13	−1.25 to −1.01	−0.25	−0.29 to −0.20
24–35 months	East Asia and the Pacific	−1.20	−1.65 to −0.74	−1.14	−1.55 to −0.73	−0.06	−0.12 to 0.01
	Eastern and Southern Africa	−1.74	−1.88 to −1.60	−1.59	−1.74 to −1.44	−0.17	−0.21 to −0.12
	Eastern Europe and Central Asia	−0.26	−0.46 to −0.07	−0.34	−0.53 to −0.14	0.07	0.00 to 0.15
	Latin America and Caribbean	−0.85	−1.18 to −0.52	−0.79	−1.09 to −0.50	−0.03	−0.10 to 0.05
	Middle East and North Africa	−1.01	−1.61 to −0.40	−0.95	−1.51 to −0.40	−0.06	−0.13 to 0.01
	South Asia	−1.58	−1.86 to −1.29	−1.56	−1.86 to −1.26	−0.03	−0.10 to 0.03
	West and Central Africa	−1.63	−1.79 to −1.47	−1.41	−1.55 to −1.26	−0.22	−0.25 to −0.18
36–47 months	East Asia and the Pacific	−1.23	−1.70 to −0.76	−1.19	−1.65 to −0.72	−0.04	−0.12 to 0.03
	Eastern and Southern Africa	−1.64	−1.81 to −1.48	−1.51	−1.67 to −1.35	−0.14	−0.19 to −0.09
	Eastern Europe and Central Asia	−0.29	−0.49 to −0.10	−0.32	−0.57 to −0.07	0.08	0.00 to 0.16
	Latin America and Caribbean	−0.81	−1.12 to −0.51	−0.80	−1.13 to −0.48	0.02	−0.04 to 0.07
	Middle East and North Africa	−0.95	−1.58 to −0.33	−1.03	−1.60 to −0.46	0.07	−0.02 to 0.15
	South Asia	−1.51	−1.84 to −1.18	−1.57	−1.91 to −1.23	0.06	0.01 to 0.10
	West and Central Africa	−1.47	−1.64 to −1.30	−1.36	−1.52 to −1.21	−0.10	−0.13 to −0.07
48–59 months	East Asia and the Pacific	−1.10	−1.55 to −0.65	−1.15	−1.61 to −0.69	0.06	0.01 to 0.11
	Eastern and Southern Africa	−1.44	−1.60 to −1.27	−1.38	−1.54 to −1.22	−0.06	−0.11 to −0.02
	Eastern Europe and Central Asia	−0.19	−0.42 to 0.04	−0.36	−0.59 to −0.14	0.18	0.12 to 0.24
	Latin America and Caribbean	−0.67	−0.99 to −0.36	−0.72	−1.04 to −0.40	0.05	0.00 to 0.10
	Middle East and North Africa	−0.88	−1.44 to −0.31	−0.96	−1.48 to −0.44	0.08	0.00 to 0.16
	South Asia	−1.45	−1.78 to −1.11	−1.48	−1.80 to −1.16	0.09	0.06 to 0.11
	West and Central Africa	−1.30	−1.46 to −1.14	−1.26	−1.41 to −1.10	−0.02	−0.06 to 0.01

In table 3, we present the number of countries with significant and non-significant sex differences in mean HAZ. For all under-5, the mean HAZ was lower for boys than for girls in 72 of the 87 countries, with statistical evidence of a difference in 51 (70.8%) of these. In the remaining 15 countries, the mean HAZ was lower in girls than boys, but no significant differences were observed. These countries where mean HAZ for girls was lower than for boys included 9 out of the 14 countries in Eastern Europe and Central Asia, 3 out of 7 in the Middle East and North Africa, 1 out of 6 in South Asia and 2 out of 12 in Latin America and the Caribbean.

**Table 3:** Number of countries according to the existence of sex differences in mean height-for-age z-scores (HAZ) by age group

Age groups	Lower HAZ among boys			Lower HAZ among girls		
	Total	Significant difference*		Total	Significant difference*	
		N	%		N	%
Total (0–59 months)	72	51	72.2	15	0	0.0
0–11 months	77	30	38.9	10	0	0.0
12–23 months	78	51	65.4	9	0	0.0
24–35 months	61	24	39.3	26	0	0.0
36–47 months	53	11	20.7	34	5	14.7
48–59 months	37	1	2.7	50	9	18.0

*Statistical significance assessed by linear regression, p<0.05.

When looking at differences according to age groups, the number of countries with male disadvantage decreased as children got older, whereas the number with female disadvantage increased with age (table 3). However, the statistically significant female disadvantage was only observed among children aged 36–47 months and 48–59 months.

**Figure 2:**
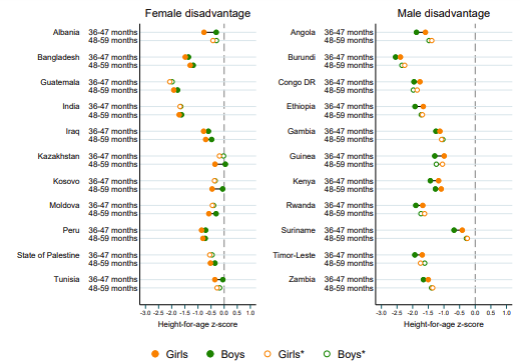
Mean height-for-age z-scores by sex in countries with (left) significant female disadvantage among children aged 36–47 months and 48–59 months and (right) significant male disadvantage among children aged 36–47 months and 48–59 months. *Non-significant difference between boys and girls.

As shown in figure 2, 11 countries presented significantly lower mean HAZ for girls among 3-year-old and 4-year-old children, namely Albania, Bangladesh, Guatemala, India, Iraq, Kazakhstan, Kosovo, Moldova, Peru, State of Palestine, and Tunisia. On the other hand, there were 11 countries with a significant male disadvantage at 36–47 months (Angola, Burundi, Democratic Republic of Congo, Ethiopia, Gambia, Guinea, Kenya, Rwanda, Suriname, Timor-Leste and Zambia), and only one wsignificant male disadvantage at both 36–47 months and 48–59 months (Kenya), as also presented in figure 2. In online supplemental efigure 1, we present sex differences by age groups for each of the 87 countries analysed.

**Figure 3:**
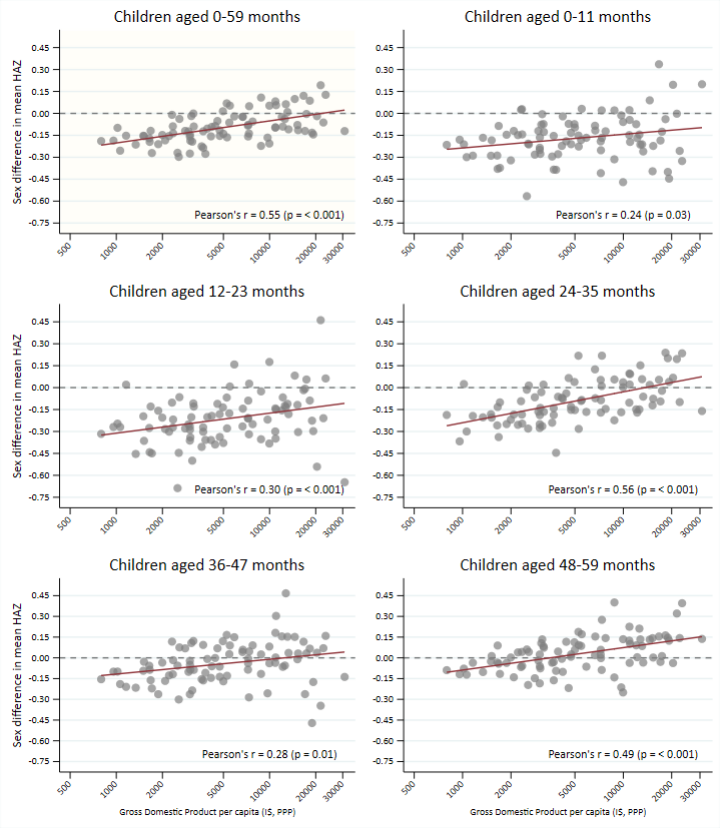
Correlation between gross domestic product per capita (international dollar, PPP, logged) and sex difference in mean height-for-age z-scores (boys minus girls) by age groups. HAZ: height-for-age z-score.

Lastly, we examined whether age-specific and sex-specific differences in mean HAZ (boys minus girls) were associated with national GDP per capita. We used the GDP per capita as a summary measure for the economic and development level of the place in which children live. Figure 3 shows that in every age group, male disadvantage tended to decrease with increasing GDP (p≤0.03 in all groups). In addition, the regression line intercepts increased with age, confirming that in many countries disadvantage for boys was no longer present among 4-year-old children, particularly in wealthier countries (online supplemental etable 3 shows regression coefficients and p values). Online supplemental efigure 2 shows the HAZ by age and sex curves for countries according to quintiles of GDP per capita, confirming the importance of national wealth in growth faltering and, also, showing that sex differences in early growth are most marked in the poorest countries (poorest quintile I$797–I$2339; second quintile I$2348–I$3691; middle quintile I$3817–I$7233; fourth quintile I$7244–I$12 592; richest quintile I$12 771–I$30 888).

## Discussion

Using a large cross-sectional dataset of children from 87 LMICs, we explored sex differences in linear growth patterns throughout the first 5 years of life. Our results confirm earlier reports showing that relative faltering occurs mainly during the first 2 years, after which mean HAZ remains roughly parallel to the median of the growth standard.^18^ In addition, we show that boys tend to be shorter relative to the international standards than girls, particularly during the first 24 months. Thereafter, however, the male disadvantage was gradually reduced and disappeared around the age of 40 months in the pooled analyses, when growth faltering became more common among girls than boys.

Our results for all under-5 children corroborate previous findings on sex differentials in stunting prevalence, which is usually higher among boys than girls.^9 13 19^ Nevertheless, we show that sex differences in linear growth vary with age, a finding that had been reported in Senegal^13^ but not in a study of 10 countries from sub-Saharan Africa.^19^ To our knowledge, ours is the first multicountry study to report that sex-specific patterns of linear growth vary with the age of children. By including 87 of the 137 LMICs in the world, our is, so far, the most representative set of analyses available in the literature.

The relative disadvantage of young boys is in agreement with existing evidence on male vulnerability, especially during infancy,^20^ and may be explained at least in part as being due to immunological factors leading to higher incidence and severity of many infectious diseases^21^ as well as sex-specific hormones, which cause higher male susceptibility to undernutrition below the age of 30 months.^12^ Hormonal factors include changing levels of testosterone throughout childhood, the dynamics of luteinising hormone and follicle-stimulating hormone, and the effect of Sertoli cell hormones, all of which show different sex patterns that may be related to linear growth faltering and occurrence of malnutrition.^12^

The higher prevalence of undernutrition among boys may be one of the pathways for their higher mortality, given that undernutrition accounts for 45% of deaths of under-5 children globally.^22^ A recent study of sex-specific mortality in 80 LMICs^23^ confirmed the well-known male disadvantage in child survival.^24 25^

When interpreting the present findings, it is important to note that z-scores are calculated through a comparison with the WHO Growth Standards, which are based on samples of children growing up under optimal conditions.^26^ The interpretation of our results, thus, refers to the differences in linear growth among boys and girls from LMICs—where growth is restricted by suboptimal nurturing care^27^—and populations with optimal growth patterns for both groups. Essentially, our results showed that the impact of suboptimal conditions in LMICs seemed to be more harmful to boys than to girls.

In turn, our finding that early male disadvantage is more marked in low-GDP countries than in wealthier countries supports the notion that under optimal environmental conditions—prevalent in higher-income countries—boys and girls would grow as well as the children on which the WHO standards were based. Although we did not include high-income countries in our analyses due to lack of standardised surveys, results for upper-middle-income countries with per capita GDP above US$12 770 show that boys and girls tend to grow in parallel with the WHO standards, only about 0.5 SD below the median value.

Although the male disadvantage in linear growth was reversed at around 36 months of age in most countries, only 11 of them showed significant female disadvantage at 4 years: 4 out of 14 from Eastern Europe and Central Asia (Albania, Kazakhstan, Kosovo, Moldova), 3 out of 7 from the Middle East and North Africa (Iraq, State of Palestine, Tunisia), 2 out of 6 from South Asia (Bangladesh, India), and 2 out of 12 from Latin America and the Caribbean (Guatemala, Peru).

Gender bias against girls leading to lower healthcare utilisation, poorer nutrition and higher than expected mortality has been described by national or local studies in several Middle Eastern and South Asian countries where we identified male nutritional advantage in older children.^28–33^ Also, widespread son preference and gender-biased sex selection have been reported in parts of Eastern Europe and Central Asia since the early 1990s.^34^ Thus, gender bias could explain why, once the early-life frailty of boys is overcome, these countries show better linear growth for boys than for girls after 3 years of age.

Significant male advantage among older children was not present in any of the 39 countries from Sub-Saharan Africa, where there is also no evidence of gender bias in under-5 mortality.^35^ Consistently, 9 of the 11 countries where male disadvantage remained significant for children aged over 36 months are from sub-Saharan Africa. The male disadvantage in nutrition has been consistently reported from this part of the world,^19 35^ even in the absence of sex differences in preventive and curative health practices, such as breast feeding, vaccination and oral rehydration therapy.^35^ These results reinforce the hypothesis that early-life sex differences in linear growth are primarily due to biological factors, with boys being more vulnerable than girls.

Our study limitations include the fact that surveys were spread over ten years, age rounding and heaping (which may explain the ‘bumps’ in HAZ curves), and the difficulties in measuring length among infants. However, to affect our conclusions such errors would have to be differential for boys and girls. The same applies to our inability to take gestational age into account in the analyses of growth in the first months of life, due to a lack of reliable data on preterm delivery. Lastly, we are inferring growth patterns from cross-sectional data in which each child was measured only once, which is a limitation affecting similar analyses of growth faltering patterns.^18^

Survival bias could also affect our interpretation. Given that undernourished children are more likely to die than those who are well nourished, and that mortality is higher for boys than for girls, surviving boys at 3 or 4 years of age may be better off in anthropometric terms than girls, partly explaining the inversion of the pattern. However, at least in some countries—such as those in Eastern Europe and Central Asi —mortality differences between under-5 boys and girls are too small in absolute terms for survival bias to affect our conclusions. In addition, the reversal from male to female disadvantage occurs at an age where mortality is low relative to that of younger children.

The strengths of our analyses comprise a large number of countries included and the high comparability among the surveys in terms of measurement protocols, sampling designs, and questionnaires. With over 800 000 children from 87 countries, this is the largest set of analyses on sex differences in linear growth of young children, allowing analyses by narrow age groups. In addition, rather than focusing on stunting prevalence that reflects children at the bottom part of the height-for-age distribution (HAZ ≤2 SD), we reported on mean HAZ that includes all children in the sample.

In conclusion, we were able to describe the patterns and magnitude of sex differences in growth faltering in children under the age of 5 years, providing evidence of male disadvantage in the first 2 years of life, followed by similar patterns for both boys and girls, or even female disadvantage at latter ages. Sex-specific vulnerability to undernutrition is rarely a consideration in the design of nutrition programmes or for the formulation of policies.^9^ Our findings are of special importance for policies and programmes targeting children’s well-being and survival, therefore, we reinforce the need for sex- and age-disaggregated analyses and reporting of nutrition estimates, which may lead to sex-specific interventions in national contexts, focusing on strategies that take into account the multifactorial nature of malnutrition. These should consider biological, social and economic aspects that might influence the child’s opportunity to thrive.

## Data Availability

Data are available in a public, open access repository. The data used are publicly available and can be downloaded free of charge for research purposes on the corresponding websites.
