# Supplementary material for: Growth patterns by sex and age among under-5 children from 87 low-income and middle-income countries
Source: BMJ Glob Health. 2021 Nov 30;6(11):e007152. doi: 10.1136/bmjgh-2021-007152 (PMC8634205; doi:10.1136/bmjgh-2021-007152)
Supplement: Supplementary data [file bmjgh-2021-007152supp001.pdf]

Costa et al. Growth patterns by sex and age among under-five children from 87 low- and middle-income countries.

Supplementary material

Costa et al. Growth patterns by sex and age among under-five children from 87 low- and middle-income countries.

eTable 1 – List of countries analyzed and corresponding survey years, data sources, and sample sizes.

country	year st	source	Ntotal
Albania	2017	DHS	2610
Algeria	2012	MICS	13726
Angola	2015	DHS	7652
Armenia	2015	DHS	1593
Bangladesh	2019	MICS	22097
Belize	2015	MICS	2418
Benin	2017	DHS	13243
Bhutan	2010	MICS	5838
Bosnia and Herzegovina	2011	MICS	2153
Burkina Faso	2010	DHS	6948
Burundi	2016	DHS	6361
CAR	2010	MICS	10243
Cambodia	2014	DHS	4906
Cameroon	2018	DHS	5167
Chad	2014	DHS	11036
Comoros	2012	DHS	2869
Congo Brazzaville	2014	MICS	8729
Congo Democratic Republic	2017	MICS	20885
Costa Rica	2018	MICS	3207
Cote d'Ivoire	2016	MICS	8949
Egypt	2014	DHS	14678
El Salvador	2014	MICS	7225
Eswatini	2014	MICS	2638
Ethiopia	2016	DHS	9626
Gabon	2012	DHS	4143
Gambia	2018	MICS	9718
Georgia	2018	MICS	2022
Ghana	2017	MICS	8797
Guatemala	2014	DHS	12280
Guinea	2018	DHS	4061
Guinea Bissau	2018	MICS	7401
Guyana	2014	MICS	2997
Haiti	2016	DHS	6769
Honduras	2011	DHS	10953
India	2015	DHS	240098
Iraq	2018	MICS	16355
Kazakhstan	2015	MICS	5263
Kenya	2014	DHS	20750
Kiribati	2018	MICS	2143
Kosovo	2013	MICS	1510
Kyrgyzstan	2018	MICS	3436
Lao	2017	MICS	11346
Lesotho	2018	MICS	3141
Liberia	2013	DHS	3863
Madagascar	2018	MICS	12444
Malawi	2015	DHS	5726
Maldives	2016	DHS	2528
Mali	2018	DHS	9521
Mauritania	2015	MICS	10058
Mexico	2015	MICS	7855
Moldova	2012	MICS	1669
Mongolia	2018	MICS	5932
Montenegro	2018	MICS	786
Myanmar	2015	DHS	4636
Namibia	2013	DHS	2643
Nepal	2016	DHS	2445
Niger	2012	DHS	5349
Nigeria	2018	DHS	12391
North Macedonia	2018	MICS	1257
Pakistan	2017	DHS	4229
Papua New Guinea	2016	DHS	4131
Paraguay	2016	MICS	4419
Peru	2018	DHS	23313
Rwanda	2014	DHS	3816
Sao Tome and Principe	2014	MICS	1944
Senegal	2017	DHS	12055
Serbia	2019	MICS	1190
Sierra Leone	2017	MICS	11399
South Africa	2016	DHS	1484
South Sudan	2010	MICS	6073
State of Palestine	2014	MICS	6939
Sudan	2014	MICS	12422
Suriname	2018	MICS	3390
Tajikistan	2017	DHS	6037
Tanzania	2015	DHS	10194
Thailand	2019	MICS	12583
Timor-Leste	2016	DHS	6667
Togo	2017	MICS	4908
Tonga	2019	MICS	1295
Trinidad and Tobago	2011	MICS	1085
Tunisia	2018	MICS	3287
Turkey	2013	DHS	2792
Turkmenistan	2019	MICS	3644
Uganda	2016	DHS	5243
Yemen	2013	DHS	14371
Zambia	2018	DHS	9617
Zimbabwe	2019	MICS	5965

Costa et al. Growth patterns by sex and age among under-five children from 87 low- and middle-income countries.

eTable 2 – Predicted mean height-for-age z-scores and 95% confidence intervals by age in months and sex at the national level based on local polynomial smoothing.

Age in months*	Boys	Girls
0.0	-0.57 (-0.63; -0.52)	-0.42 (-0.48; -0.36)
1.2	-0.58 (-0.62; -0.53)	-0.40 (-0.45; -0.35)
2.4	-0.59 (-0.63; -0.54)	-0.41 (-0.45; -0.37)
3.6	-0.57 (-0.62; -0.53)	-0.43 (-0.47; -0.39)
4.8	-0.57 (-0.61; -0.52)	-0.47 (-0.51; -0.43)
6.0	-0.59 (-0.63; -0.55)	-0.48 (-0.52; -0.44)
7.2	-0.67 (-0.71; -0.62)	-0.52 (-0.56; -0.48)
8.4	-0.78 (-0.83; -0.74)	-0.56 (-0.60; -0.52)
9.6	-0.90 (-0.95; -0.85)	-0.65 (-0.69; -0.61)
10.8	-1.01 (-1.05; -0.96)	-0.74 (-0.78; -0.70)
12.0	-1.11 (-1.16; -1.06)	-0.84 (-0.88; -0.80)
13.2	-1.22 (-1.26; -1.17)	-0.96 (-1.01; -0.92)
14.4	-1.32 (-1.36; -1.27)	-1.08 (-1.13; -1.04)
15.7	-1.40 (-1.45; -1.36)	-1.18 (-1.22; -1.13)
16.9	-1.48 (-1.53; -1.44)	-1.25 (-1.29; -1.20)
18.1	-1.54 (-1.59; -1.49)	-1.33 (-1.38; -1.29)
19.3	-1.59 (-1.64; -1.54)	-1.42 (-1.47; -1.37)
20.5	-1.63 (-1.68; -1.58)	-1.48 (-1.53; -1.43)
21.7	-1.66 (-1.71; -1.61)	-1.49 (-1.54; -1.44)
22.9	-1.65 (-1.70; -1.60)	-1.46 (-1.50; -1.41)
24.1	-1.60 (-1.65; -1.55)	-1.40 (-1.45; -1.35)
25.3	-1.54 (-1.59; -1.49)	-1.36 (-1.4; -1.31)
26.5	-1.53 (-1.59; -1.48)	-1.38 (-1.43; -1.33)
27.7	-1.56 (-1.61; -1.50)	-1.43 (-1.48; -1.38)
28.9	-1.60 (-1.66; -1.55)	-1.49 (-1.54; -1.44)
30.1	-1.64 (-1.70; -1.58)	-1.52 (-1.57; -1.47)
31.3	-1.63 (-1.69; -1.57)	-1.52 (-1.57; -1.48)
32.5	-1.61 (-1.67; -1.55)	-1.54 (-1.59; -1.49)
33.7	-1.59 (-1.65; -1.54)	-1.55 (-1.60; -1.50)
34.9	-1.57 (-1.62; -1.52)	-1.51 (-1.56; -1.46)
36.1	-1.53 (-1.58; -1.48)	-1.46 (-1.50; -1.41)
37.3	-1.54 (-1.58; -1.49)	-1.42 (-1.47; -1.38)
38.5	-1.54 (-1.60; -1.49)	-1.46 (-1.51; -1.41)
39.7	-1.54 (-1.59; -1.48)	-1.51 (-1.56; -1.46)
40.9	-1.55 (-1.60; -1.49)	-1.54 (-1.59; -1.49)
42.1	-1.58 (-1.63; -1.52)	-1.55 (-1.60; -1.51)
43.3	-1.59 (-1.65; -1.53)	-1.56 (-1.61; -1.51)
44.6	-1.58 (-1.64; -1.52)	-1.55 (-1.61; -1.50)
45.8	-1.54 (-1.59; -1.49)	-1.55 (-1.60; -1.50)
47.0	-1.47 (-1.52; -1.42)	-1.49 (-1.55; -1.44)
48.2	**-1.38 (-1.44; -1.33)**	**-1.45 (-1.50; -1.40)**
49.4	**-1.35 (-1.40; -1.30)**	**-1.42 (-1.46; -1.37)**
50.6	**-1.37 (-1.42; -1.32)**	**-1.43 (-1.48; -1.38)**
51.8	**-1.40 (-1.45; -1.35)**	**-1.44 (-1.48; -1.39)**

Costa et al. Growth patterns by sex and age among under-five children from 87 low- and middle-income countries.

Age in months*	Boys	Girls
53.0	**-1.41 (-1.46; -1.36)**	**-1.43 (-1.47; -1.39)**
54.2	**-1.41 (-1.46; -1.36)**	**-1.44 (-1.48; -1.40)**
55.4	**-1.41 (-1.47; -1.36)**	**-1.48 (-1.52; -1.43)**
56.6	**-1.43 (-1.49; -1.38)**	**-1.49 (-1.54; -1.44)**
57.8	**-1.46 (-1.52; -1.40)**	**-1.50 (-1.55; -1.45)**
59.0	**-1.48 (-1.55; -1.41)**	**-1.49 (-1.55; -1.42)**

Note: gray cells represent overlapping confidence intervals; bold text represents lower absolute HAZ among girls
* Age values at which the local polynomial smoothing was calculated.

Costa et al. Growth patterns by sex and age among under-five children from 87 low- and middle-income countries.

eFigure 1 – Sex difference in mean height-for-age z-scores (boys minus girls) by country and age groups. Countries are ordered from the lowest to the highest difference value. Negative values indicate that boys fared worse than girls.

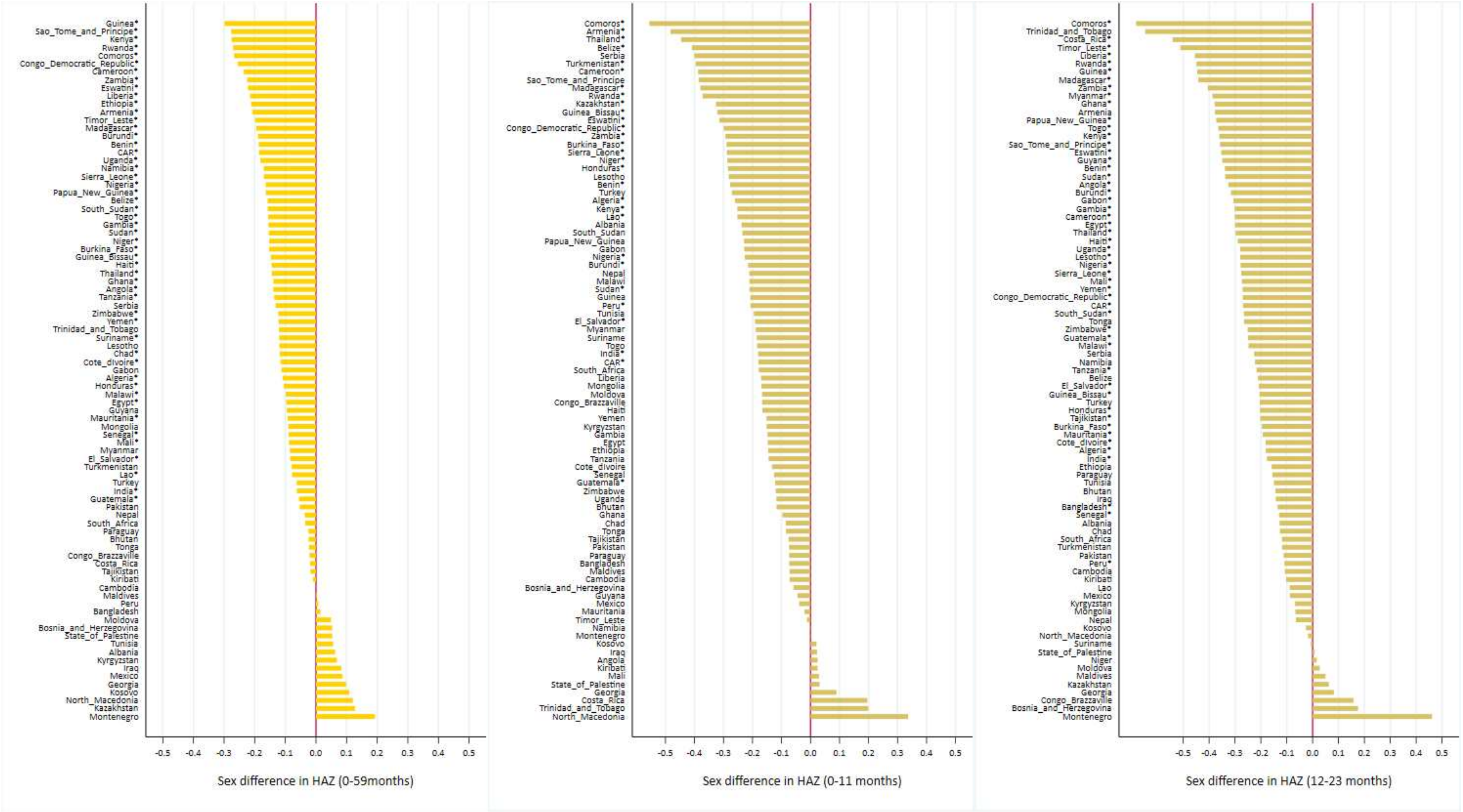

Costa et al. Growth patterns by sex and age among under-five children from 87 low- and middle-income countries.

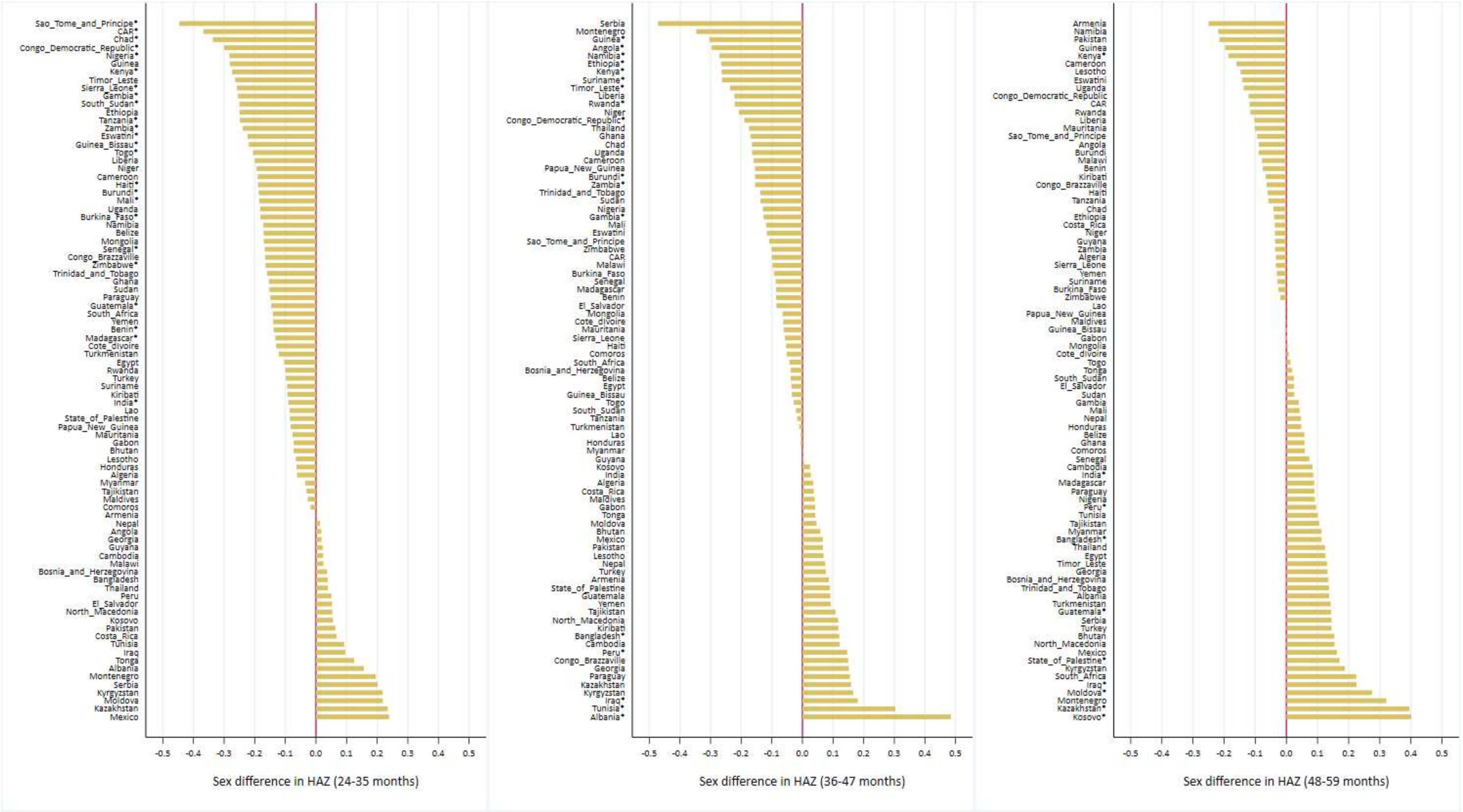

* Statistically significant sex differences

Costa et al. Growth patterns by sex and age among under-five children from 87 low- and middle-income countries.

eTable 3 – Linear regression coefficients for the association between sex differences in height-for-age z-scores (boys minus girls) and log-transformed GDP per capita.

Age group	Intercept	Slope	p value
Total (0-59 months)	-0.65	0.07	<0.0001
0-11 months	-0.52	0.04	0.03
12-23 months	-0.72	0.06	<0.0001
24-35 months	-0.87	0.09	<0.0001
36-47 months	-0.44	0.05	0.01
48-59 months	-0.58	0.07	<0.0001

Costa et al. Growth patterns by sex and age among under-five children from 87 low- and middle-income countries.

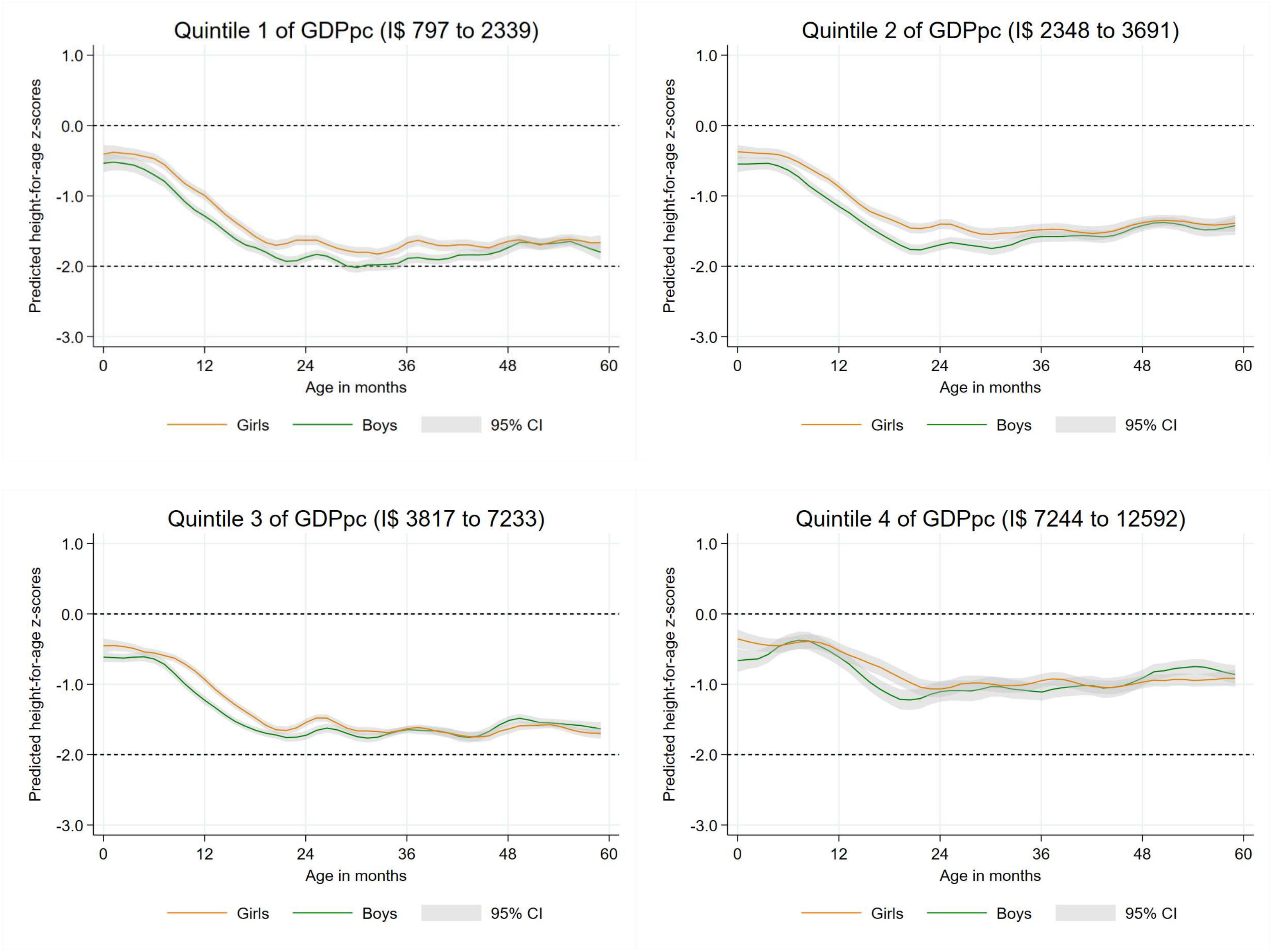

Costa et al. Growth patterns by sex and age among under-five children from 87 low- and middle-income countries.

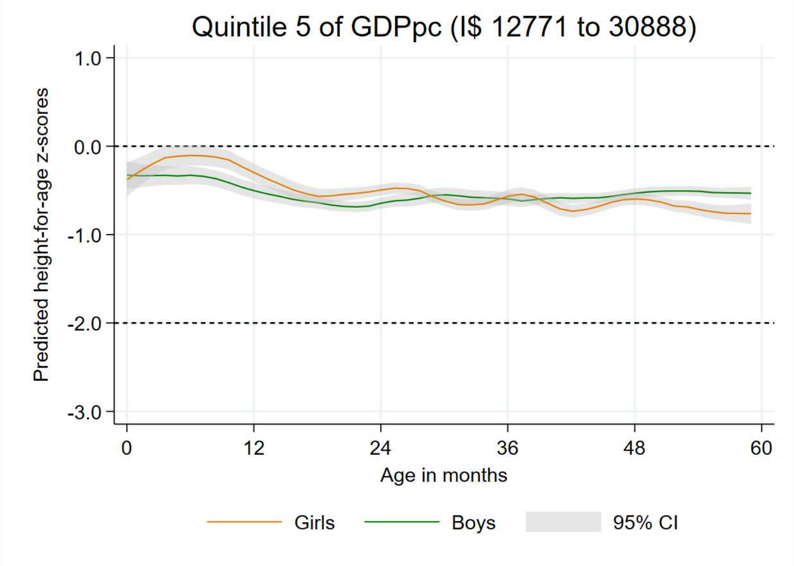

eFigure 2 – Local polynomial predicted curves for mean height-for-age z-scores according to age in months by sex. Pooled results for under-five children from low- and middle-income countries divided into quintiles of GDP per capita (in current international dollars – I$).
